# Fibrinogen and D-dimer alterations in thyroid dysfunction: implications for bleeding and thrombotic risk

**DOI:** 10.1186/s13044-026-00312-8

**Published:** 2026-07-27

**Authors:** Befikad Mandefro, Amanuel Kelem, Tiruneh Adan

**Affiliations:** 1 Department of Medical Laboratory Sciences, College of Medicine and Health Sciences, Debre Berhan University, Debre Berhan, Ethiopia; 2 Department of Hematology and Immunohematology, School of Biomedical and Laboratory Sciences, College of Medicine and Health Sciences, University of Gondar, Gondar, Ethiopia

**Keywords:** Fibrinogen, D-dimer, Fibrinolysis, Thyroid dysfunction, Bleeding, Venous thrombosis

## Abstract

Thyroid dysfunction can disrupt the balance of the hemostatic system, predisposing affected individuals to either bleeding or thrombotic complications, depending on the nature and severity of the disorder. In hyperthyroidism, a prothrombotic state is commonly observed, characterized by increased fibrinogen concentrations, elevated D-dimer levels, and enhanced coagulation activation. Conversely, hypothyroidism may impair coagulation and fibrinolysis, resulting in variable bleeding or thrombotic manifestations depending on disease severity. Raised D‑dimer levels reflect increased thrombin generation with secondary fibrinolysis and therefore serve as markers of a hypercoagulable state. Fibrinogen concentrations above 4.0 g/L are independently associated with a higher risk of venous thromboembolism, although a direct causal role has not been established. In patients with hyperthyroidism, fibrinogen levels are increased by approximately 20% on average, suggesting an elevated thrombotic risk, potentially mediated by reduced fibrinolytic capacity. Hyperthyroid patients consistently demonstrate higher plasma D‑dimer concentrations, indicating concurrent activation of coagulation and fibrinolysis. Previous studies have documented increased D‑dimer levels in both overt and subclinical hyperthyroidism, consistent with upregulation of the fibrinolytic pathway. D‑dimer values in the range of approximately 450–650 ng/mL are frequently observed in this population. In moderate hypothyroidism, relatively lower D‑dimer levels have been linked to diminished fibrinolytic activity. Bleeding manifestations have been frequently reported in patients with hypothyroidism, although their prevalence varies considerably depending on the study population and clinical criteria used. In contrast, thrombotic complications are more commonly associated with hyperthyroidism, with reported frequencies varying widely across studies due to differences in disease severity, population characteristics, and outcome definitions. In summary, thyroid dysfunction disturbs hemostatic equilibrium and leads to characteristic changes in fibrinogen and D‑dimer levels. Abnormal concentrations of these markers may signal impaired hemostatic function and could help identify patients at increased risk of bleeding or thrombosis.

## Introduction

Hemostasis is a tightly regulated physiological process that maintains vascular integrity by preventing excessive bleeding while allowing controlled thrombus formation following vascular injury [1]. It involves a coordinated interaction between the vascular endothelium, platelets, coagulation factors, anticoagulant pathways, and the fibrinolytic system to ensure effective clot formation and subsequent clot resolution [2, 3]. Disruption of this finely balanced system may result in either bleeding or thrombotic complications. Endocrine disorders, particularly thyroid dysfunction, are recognized causes of hemostatic abnormalities, ranging from mild laboratory coagulation changes with limited clinical significance to clinically important bleeding and thromboembolic events [4]. Thyroid hormones play a central role in regulating numerous physiological and metabolic processes, including lipid metabolism, cell proliferation, mitochondrial respiration, and the hemostatic system [5–7]. Their effects on coagulation and fibrinolysis are mediated through both thyroid hormone receptor-dependent genomic pathways and receptor-independent mechanisms [8]. Consequently, both excess and deficiency of thyroid hormones can disrupt the balance between coagulation and fibrinolysis, thereby increasing the risk of either thrombosis or bleeding.

Several studies have shown that hypothyroidism is associated with an increased bleeding tendency due to abnormalities in both primary and secondary hemostasis [9]. In contrast, hyperthyroidism is generally associated with a hypercoagulable state characterized by increased levels of procoagulant and antifibrinolytic factors, including fibrinogen and plasminogen activator inhibitor-1 (PAI-1), which promote thrombus formation [10]. Alterations in plasma fibrinogen and D-dimer concentrations are among the most consistently reported hemostatic abnormalities in patients with thyroid dysfunction [11]. In particular, free thyroxine (FT4) has been positively associated with plasma fibrinogen levels, suggesting that thyroid hormone status directly influences coagulation activity [12].

Patients with overt hyperthyroidism exhibit significantly higher plasma fibrinogen concentrations than euthyroid individuals, indicating enhanced coagulation activity and reduced fibrinolytic capacity [13]. Similar findings have been reported in patients with subclinical hyperthyroidism, supporting the presence of a prothrombotic state even in the absence of overt clinical manifestations [8, 13–16]. Elevated fibrinogen concentrations (> 4.0 g/L) are independently associated with an increased risk of venous thromboembolism, although fibrinogen itself is considered a marker rather than a direct cause of thrombosis. Likewise, elevated D-dimer levels reflect increased thrombin generation, fibrin formation, and secondary fibrinolysis, making D-dimer a reliable biomarker of ongoing coagulation activation and fibrin turnover. Therefore, increased D-dimer concentrations in hyperthyroidism are more indicative of a hypercoagulable state than isolated activation of fibrinolysis [8, 14–16].

Conversely, patients with hypothyroidism often exhibit lower fibrinogen concentrations and reduced coagulation activity, which may contribute to an increased bleeding tendency [17]. Moderate hypothyroidism has also been associated with lower D-dimer levels, reflecting reduced fibrin turnover and fibrinolytic activity. In contrast, D-dimer concentrations are consistently elevated in patients with overt hyperthyroidism compared with healthy individuals and generally decline following restoration of euthyroidism, indicating that these alterations are largely reversible with appropriate treatment [11, 13, 18]. Overall, changes in fibrinogen and D-dimer levels reflect alterations in coagulation activation and fibrin turnover and may serve as useful biomarkers for assessing the risk of bleeding and thrombotic complications in patients with thyroid dysfunction [19].

Despite growing evidence linking thyroid dysfunction with hemostatic abnormalities, the reported findings remain heterogeneous, and the clinical significance of fibrinogen and D-dimer alterations has not been comprehensively summarized. Therefore, this review aims to critically evaluate the available evidence on changes in fibrinogen and D-dimer levels across different thyroid disorders and to discuss their potential implications for bleeding and thrombotic risk.

### Mechanism of hemostasis disorder in thyroid hormone imbalance

Thyroid hormones are important regulators of hemostatic balance through coordinated effects on coagulation, anticoagulation, fibrinolysis, platelet function, and endothelial activity. These effects are mediated through two major pathways: genomic and non-genomic mechanisms. In the genomic pathway, thyroid hormones bind to nuclear thyroid hormone receptors and regulate the transcription of target genes involved in hepatic synthesis of coagulation proteins, anticoagulant factors, and fibrinolytic regulators. Therefore, alterations in thyroid hormone levels can modify the synthesis, regulation, and clearance of multiple hemostatic proteins, resulting in disruption of the physiological equilibrium between coagulation and fibrinolysis [7].

Thyroid hormone imbalance affects hemostasis through multiple interconnected mechanisms involving coagulation factors, platelets, endothelial cells, and fibrinolytic pathways. Changes in hepatic protein synthesis may alter concentrations of coagulation factors, including fibrinogen and factor VIII (FVIII), as well as endogenous anticoagulant proteins. These abnormalities can shift the hemostatic system toward either excessive bleeding or a hypercoagulable state depending on the type and severity of thyroid dysfunction.

In addition to genomic regulation, thyroid hormones exert rapid non-genomic effects on vascular and cellular components of hemostasis. Thyroid hormones influence platelet activation and endothelial responsiveness by modifying intracellular signaling pathways. Increased thyroid hormone activity enhances tissue sensitivity to catecholamines, which may stimulate endothelial release of von Willebrand factor (VWF), promoting platelet adhesion and primary hemostasis. Furthermore, thyroid hormones regulate endothelial nitric oxide synthase (eNOS) and prostacyclin pathways, and disturbances in these pathways may impair vasodilation and contribute to endothelial dysfunction and a prothrombotic vascular environment [13, 20, 21].

Thyroid dysfunction also modifies fibrinolytic activity, which determines the removal of fibrin clots. Enhanced fibrinolysis due to increased fibrinolytic mediators or reduced antifibrinolytic activity may contribute to bleeding tendency. However, increased expression of plasminogen activator inhibitor-1 (PAI-1), a major inhibitor of fibrinolysis, reduces plasmin generation and promotes fibrin persistence, thereby favoring thrombus formation [22].

Multiple hemostatic abnormalities involving platelets, coagulation factors, cytokines, and endothelial pathways have been reported in patients with thyroid disorders [20, 23]. In hypothyroidism, reduced thyroid hormone stimulation may decrease hepatic synthesis and turnover of certain coagulation proteins. Altered adrenergic responsiveness and endothelial dysfunction may further influence coagulation factor activity, particularly FVIII and VWF [24–26]. Although some studies have reported reduced fibrinogen and D-dimer levels, suggesting reduced coagulation activation, hypothyroidism may simultaneously increase cardiovascular risk through inflammation, dyslipidemia, and endothelial dysfunction.

In contrast, hyperthyroidism is generally associated with a hypercoagulable state characterized by increased synthesis and activity of coagulation proteins, enhanced platelet activation, increased endothelial release of VWF, and increased fibrin turnover. Elevated fibrinogen and D-dimer concentrations in hyperthyroid patients reflect enhanced coagulation activation and fibrin degradation, which may contribute to an increased risk of venous and arterial thrombotic complications [27].

In general, thyroid hormones maintain hemostatic equilibrium by integrating hepatic coagulation protein synthesis, platelet responsiveness, endothelial function, and fibrinolytic regulation. Disturbance of thyroid hormone homeostasis disrupts this balance and produces a spectrum of hemostatic abnormalities ranging from bleeding manifestations to increased thrombotic risk (Table 1) [27].

**Table 1:** Mechanism and effects of hypothyroid and hyperthyroidism on hemostasis

Thyroid dysfunction	Effects on hemostasis	Proposed mechanism	Level of evidence
Hypothyroidism	Decrease the level of D-dimer and fibrinogen Increase risk of bleeding	Hypercoagulability manly increased level of VWF and Factor VIII and impaired fibrinolysis.	Observational study on clinical end points and Interventional studies on parameters of coagulation and fibrinolysis
Hyperthyroidism	Increase the level of D-dimer and fibrinogen increase the risk of venous thrombosis (DVT, PE VTE)	Increased prevalence of acquired von will brad diseases.	Observational study on parameters of coagulation and fibrinolysis

The structure of the fibrin network influences thrombotic risk. Clots composed of thinner fibers, smaller pores, and a more compact structure are more resistant to fibrinolysis and are associated with increased risk of vascular occlusion [28]. The increased plasma fibrinogen levels may have been due to increased fibrinogen production caused by activation of liver function. The high plasma fibrinogen levels could be the result of a persistent inflammatory state brought on by thyroid inflammatory illnesses [8, 9, 16]. Thyroid-stimulating hormone has been demonstrated to contribute to endothelial dysfunction through altered regulation of genes involved in endothelial nitric oxide synthase (eNOS), prostacyclin synthesis, and other key vasoactive and hemostatic mediators [29]. The influence of thyroid hormones on the coagulation–fibrinolytic system is mediated through both genomic and nongenomic pathways; however, many of these effects appear to occur predominantly via nongenomic mechanisms that are independent of the primary interaction between triiodothyronine (T3) and nuclear thyroid hormone receptors [23, 30, 31]. Interleukin-1 (IL-1) activation can result in the creation of prothrombotic factors such as super large VWF multimers and tissue factor, as well as inflammatory cytokines such as IL-6 and IL-8 and adhesion molecules [32].

Thyroid hormone analogues such as tetraiodothyronine acetic acid and modified tetra iodothyrin acetic acid regulate interleukin-1 gene expression [33]. In both preclinical and overt hyperthyroidism, levels of pro-inflammatory cytokines related to coagulation and endothelial dysfunction favoring coagulability are present [34]. The authors demonstrated that T3-induced expression of genes responsible for the production of fibrinogen, thrombin, and other coagulation factors [35]. The T_4_-initiated mechanisms comprise platelet activation, in a D-dimertion, cellular production of cytokines and chemokines such as CX3CL1 with procoagulatory activities. T4’s procoagulant effects are especially noteworthy because T4 is not considered as a functioning hormone within cells, but rather as a prohormone for T3.

Finally, thyroid hormones may enhance platelet–endothelial cell interactions, contributing to local thrombus formation. Toll-like receptors (TLRs), key regulators of the innate immune system, are expressed not only in thyroid follicular cells but also on platelet surfaces. Notably, certain TLR polymorphisms have been associated with cardioprotective effects, as they are linked to reduced platelet thromboxane biosynthesis, diminished platelet function, and altered platelet activation, shifting their role from primarily hemostatic regulators to immune sentinels [36, 37]. In a D-dimertion, pro-inflammatory cytokines such as interleukin-1 (IL-1), interferon, and tumor necrosis factor-alpha (TNF-α) are elevated in autoimmune thyroid disorders, where they can inhibit collagen-induced platelet aggregation [23, 38–41].

### Fibrinogen level in thyroid dysfunction patient

Fibrinogen is a large glycoprotein, synthesized primarily in the liver which is the most abundant blood coagulation factor [42]. Thyroid function is linked to plasma fibrinogen level, specifically free T4, which was found to be highly linked to fibrinogen [12].

In hyperthyroidism, fibrinogen levels have been reported to increase by approximately 17%, suggesting enhanced coagulation activation and increased thrombotic risk [43]. Higher fibrinogen levels, as well as non-significant increases in PAI-1 and antiplasmin levels, are connected to hyperthyroidism. Due to greater fibrinogen levels, high levels of T4 cause hypercoagulability. A positive association between thyroid hormone activity and fibrinogen concentration has been reported in hyperthyroid patients [8, 14, 15].

Fibrinogen level was significantly increase in hyperthyroidism patients. It ranges from 2.1 to 4.6 g/l [22, 44]. This was higher than the euthyroidism and the normal reference value of fibrinogen is 2–4.45 g/L in average. This increases in mean levels of fibrinogen indicating the high increased risk of thrombosis. Decreased fibrinolytic activity is common in overt hyperthyroidism and after thyroid hormone treatment [45, 46]. In a cohort of patients with untreated hyperthyroidism and healthy controls, plasma concentrations of fibrinogen and fibrinopeptide Bβ15–42, a fibrinogen-derived product generated by plasmin-mediated cleavage, were examined. The plasma levels of both measures were significantly higher [47, 48].

On the other side, fibrinogen levels are significantly lower in hypothyroid patients which is good indicator of bleeding. In hypothyroidism, no definitive pattern of alteration in fibrinogen levels was detected in mild or moderate hypothyroidism. Its levels were significantly higher in moderate subclinical hypothyroidism (SCH) and hypothyroid groups at the initial visit. Its value ranging from 216.71 mg/dL in severe hypothyroidism to 269.96 mg/dL after attaining euthyroid state [44, 49]. Those value also decrease from euthyroid 2.42 g/l to hypothyroidism of 2.26 g/l [9, 50](Table 2).

**Table 2:** Fibrinogen level in thyroid dysfunction patient

Result		Control* or reference	Conclusion	Authors
Hypothyroidism				
Min	Max			
2.57 g/l	4.45 g/l	334 g/L ± 79*	Normal	Elbers et al., 2016 [49]
2.26 g/l	2.42 g/l	255.0 ± 41.7 g/L*	Decreased	Stuijver et al., 2012 [17]
2.3 g/l	4.11 g/l	298 g/L ± 79*	Decreased	Toruner et al., 2008 [51]
1.76 g/l	2.83 g/l	254 ± 58*g/l	Decreased	Thoyyib et al., 2018 [44].
2.8 g/L	5.23 g/L	350 ± 54 g/l*	Normal	Vayá et al., 2014)[52]
2.05 g/l	3.2 g/L	2.89 ± 0.6 g/L*	Decreased	Chadarevian et al., 2001 [53]
2.77 g/L)	3.8 g/L)	3.15 ± 0.27.1 g/L) *	Decreased	Cantürk et al., 2003 [54]

Hyperthyroidism		Reference or Control*	Conclusion	Authors
Min	Max			
2.6 g/l	6.54 g/l	189–412 g/L*	Increased	Semir et al.,2010 [55]
3.07 g/l	4.57 g/l	2.2–4.5 g/L	Increased	Brona et al., 2011 [11]
2.26 g/l	2.976 g/l	2.55.0 ± 0.42 g/L*	Increased	Coban and Aydemir, 2008 [56]
2.57 g/l	6.55 g/l	2.1–4.43 g/L*	Increased	Pasa et al., 2010 [57].

**Fig. 1:**
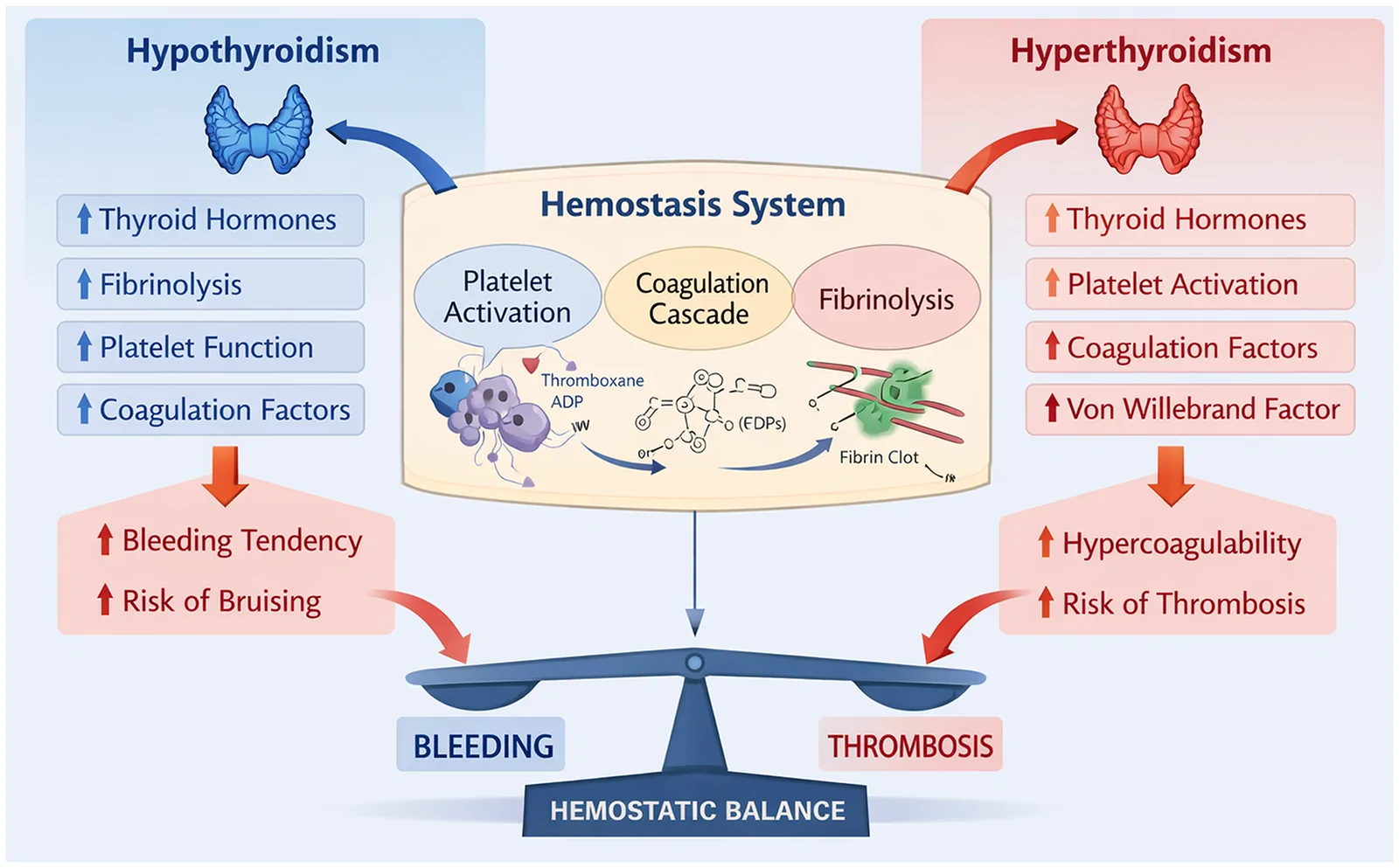
General mechanism of thyroid dysfunction on hemostasis system

As shown in Table 2, there is a progressive rise in fibrinogen levels as thyroid function shifts from hypo- to hyperthyroid states. This pattern indicates that hepatic fibrinogen synthesis is highly responsive to T₄ levels, potentially serving as a key contributor to the increased thrombotic risk observed in hyperthyroidism.High fibrinogen levels (> 4.5 g/L) can encourage thrombosis by forming denser clots that are more resistant to breakdown, whereas low levels (< 1.5 g/L) weaken normal hemostasis. Thyroid function further influences this balance: in hyperthyroidism, T3 and T4 enhance fibrinogen production through gene regulation, while in hypothyroidism, reduced synthesis is accompanied by a potential prothrombotic tendency, partly due to increased PAI-1-1 (Fig. 1).

### D-dimer level in patient with thyroid dysfunction

D-dimer is a fibrin degradation product generated during the breakdown of cross-linked fibrin and serves as a biomarker of coagulation activation and fibrin turnover. It reflects ongoing intravascular fibrin formation followed by secondary fibrinolysis and is widely used in clinical practice, particularly for the exclusion of venous thromboembolism (VTE) in patients with appropriate clinical assessment. Although D-dimer testing has high sensitivity, its clinical interpretation is limited by relatively low specificity and variability related to factors such as age, inflammation, and assay methods. Despite these limitations, D-dimer measurement remains an important tool in the evaluation of thrombotic risk and may support clinical decision-making regarding anticoagulation management [58, 59]. It is also commonly applied in the assessment of suspected pulmonary embolism (PE) and deep vein thrombosis (DVT) as part of a diagnostic strategy combined with clinical probability assessment and imaging findings [60].

D-dimer levels are frequently altered in patients with thyroid dysfunction, with abnormal concentrations reported in approximately 48.3% of affected individuals [61]. Increased D-dimer levels have been more consistently observed in patients with hyperthyroidism, with reported values ranging from 23 to 1326 ng/mL, exceeding those observed in euthyroid individuals and, in some studies, surpassing the reference range of approximately 70–490 ng/mL [11]. These elevations reflect increased thrombin generation, fibrin formation, and subsequent fibrin degradation, indicating enhanced coagulation activation and fibrinolytic activity rather than isolated fibrinolysis. The magnitude of D-dimer elevation may depend on the severity and autoimmune nature of the underlying thyroid disorder and may decrease following restoration of euthyroidism [11, 56]. Mohamed et al. (2016) reported significantly higher D-dimer levels in patients with hyperthyroidism compared with healthy controls and demonstrated a significant association between thyroid hormone levels (T3 and T4) and plasma D-dimer concentrations [18]. Furthermore, normalization of thyroid function was associated with a significant reduction in D-dimer levels, suggesting that thyroid-related alterations in coagulation and fibrinolysis are potentially reversible. However, exogenous hyperthyroidism has not consistently demonstrated similar changes in D-dimer levels, indicating possible differences between endogenous and exogenous thyroid hormone excess [28].

Severe hypothyroidism has been associated with elevated D-dimer concentrations, and free T4 shows an inverse relationship with D-dimer. These changes reflect disturbances in coagulation activation and fibrinolytic balance, serving as biochemical indicators of a prothrombotic state and thromboembolic tendency [62]. Reported D-dimer values in hypothyroid patients show variability across studies, ranging from approximately 47 to 900 mg/dL although this wide range likely reflects differences in study design and, importantly, inconsistencies in unit reporting across the literature [53]. While severe overt hypothyroidism is classically associated with a bleeding tendency, patients with mild-to-moderate hypothyroidism or subclinical disease may show a paradoxical shift toward a prothrombotic state; this hypofibrinolytic profile is reflected by reduced D-dimer levels and diminished baseline fibrin turnover. This pattern reflects diminished fibrin degradation and a shift in hemostatic balance toward a less active fibrinolytic state [48]. Moderate hypothyroidism has been associated with reduced fibrinolytic activity, as reflected by lower D-dimer levels, indicating that thyroid hormone deficiency may influence the fibrinolytic system. In contrast, elevated D-dimer levels reflect thrombin generation followed by secondary fibrinolysis rather than primary fibrinolysis, serving as an indicator of a hypercoagulable state. Similarly, fibrinogen concentrations above 4.0 g/L are independently associated with an increased risk of venous thromboembolism and serve as a biomarker of thrombotic tendency [63] (Table 3).

**Table 3:** The level of D-dimer in thyroid dysfunction patient

Result		Control* or reference	Conclusion	Authors
Hypothyroidism				
Min	Max			
47.81 ng/ml	905.81 ng/ml	311.05 ± 184 ng/Ml*	Low to high (inconsistence)	Chadarevian et al., 2001 [53]
631 ng/ml	689 ng/ml	654 ng/mL*	Low to high (Inconsistence)	Segna et al., 2016 [64]
52 ng/mL	75ng/mL	90 ± 27 ng/mL*	Low	Cantürk et al., 2003 [54]

Hyperthyroidism		Control*or reference	Conclusion	Authors
Min	Max			
200 ng/mL	**300** ng/mL	200 ± 30 ng/mL	Increased	Coban and Aydemir, 2008 [56]
21.55 ng/m	1326.97 ng/mL	70–490 ng/mL	Increased	Brona et al., 2011 [11].
646 ng/mL	696ng/mL	654 ng/mL	Increase	Segna et al., 2016) [64]
0.031 mg/dL	**0.088 mg/dL**	**0.017–0.027 mg/dL**	Increased	Elbers et al., 2016 [65]

### Thrombosis prevalence in thyroid dysfunction patients

Thyroid dysfunction, particularly hyperthyroidism, has been increasingly recognized as a condition that may influence thrombotic risk through alterations in coagulation and fibrinolytic pathways. Population-based studies have reported a higher risk of venous thromboembolism (VTE) among individuals with hyperthyroidism compared with euthyroid populations. Findings from hospital-based studies support this association, showing increased occurrences of deep vein thrombosis (DVT) and pulmonary embolism (PE) among patients with hyperthyroidism. The variation in reported risk estimates across studies may be related to differences in study populations, diagnostic approaches, disease severity, and definitions of thrombotic outcomes [21]. Individuals with a history of hyperthyroidism have been reported to have approximately a 1.6-fold increased risk of venous thrombosis compared with individuals without thyroid disease [10, 66–68]. Furthermore, a large population study involving 53,418 patients demonstrated that hyperthyroidism was associated with a 2.31-fold higher risk of pulmonary embolism compared with controls [35, 69, 70].

The available evidence indicates that excessive thyroid hormone activity may promote a prothrombotic state, increasing susceptibility to complications such as cerebral venous sinus thrombosis, DVT, and PE. Among these thrombotic outcomes, the relationship between hyperthyroidism and DVT appears to be particularly prominent, with DVT being the most commonly reported venous thrombotic event in this population [66–68, 71]. Among hyperthyroid patients experiencing thrombotic complications, 63.3% developed DVT, 30.1% developed PE, and 6.6% experienced both DVT and PE [72]. Hyperthyroidism has also been associated with arterial thrombotic events, with reported prevalence estimates ranging from approximately 8% to 40%, depending on the clinical characteristics and study population. The wide variation in reported thrombotic and bleeding events (0.7%–64%) may reflect differences in study design, patient selection, disease classification, and outcome assessment, including distinctions between overt and subclinical thyroid disorders [73, 74].

Thrombotic complications have also been reported among patients with hypothyroidism; however, the strength of this association remains less consistent compared with hyperthyroidism. The reported frequency varies considerably between studies, likely due to differences in population characteristics, disease severity, treatment status, and methodological approaches. DVT appears to be the most frequently described thrombotic event in hypothyroid patients, followed by VTE and PE. Reported DVT prevalence ranges from 2% to 53% among individuals with hypothyroidism [6], while another study documented DVT in 1.36% of hypothyroid patients [75]. The occurrence of VTE has been reported between 0.22% and 4.4% in this population [76], with one study reporting a VTE incidence of 7.2% [77]. Pulmonary embolism has been reported less frequently, occurring in approximately 0.61%–1.42% of patients with hypothyroidism [75].

### Magnitude of bleeding in thyroid dysfunction patients

Several studies have reported an increased tendency for bleeding among patients with hypothyroidism, ranging from mild mucocutaneous bleeding manifestations to more severe hemorrhagic complications following trauma or surgical procedures. Thyroid dysfunction also affects the female reproductive system, with menstrual abnormalities occurring more frequently. These hemostatic disturbances are mainly associated with abnormalities in primary hemostasis, including impaired platelet function and acquired coagulation defects, which may contribute to increased bleeding risk.

The reported frequency of bleeding manifestations varies considerably among studies, likely due to differences in study populations, diagnostic criteria, disease severity, and definitions of bleeding outcomes [4, 20, 27, 78–80]. Overall, bleeding manifestations in hypothyroidism have been reported in approximately 40%–60% of patients [78, 81]. Menorrhagia is the most frequently observed bleeding manifestation in hypothyroid patients, affecting approximately 54.54%, whereas hypomenorrhea is less commonly reported. Major bleeding events are relatively uncommon but have been documented in approximately 2.5% of individuals with hypothyroidism [82, 83].

Although less frequent than in hypothyroidism, bleeding manifestations may also occur in patients with hyperthyroidism. Menstrual disturbances are among the commonly reported presentations, with oligomenorrhea being the predominant abnormality, while metrorrhagia occurs less frequently. The reported prevalence of abnormal uterine bleeding in hyperthyroid patients ranges from approximately 1.3% to 40% [84, 85].

## Conclusion

Hemostasis is a tightly regulated process that maintains vascular integrity through the coordinated interaction of coagulation factors, anticoagulant pathways, fibrinolysis, platelets, and endothelial function. Thyroid hormones play an important role in maintaining this balance, and disturbances in thyroid function can alter coagulation and fibrinolytic pathways, leading to either bleeding or thrombotic complications.

Hyperthyroidism, including subclinical forms, is more consistently associated with a prothrombotic state characterized by increased fibrinogen and D-dimer levels, reflecting enhanced coagulation activation and fibrin turnover. These alterations may contribute to an increased risk of venous thromboembolism and may improve after restoration of euthyroidism. In contrast, hypothyroidism is associated with variable hemostatic changes, including reduced fibrinogen levels, impaired coagulation factor synthesis, altered platelet function, and disturbances in fibrinolysis, which may contribute to bleeding manifestations. However, hypothyroidism may also be associated with cardiovascular risk through inflammatory, metabolic, and endothelial abnormalities.

Generally, thyroid dysfunction significantly influences the hemostatic system through complex interactions involving coagulation proteins, fibrinolysis, platelets, and vascular function. Fibrinogen and D-dimer may serve as potential biomarkers for assessing coagulation and fibrinolytic alterations in thyroid disorders; however, their value in predicting clinical bleeding and thrombotic outcomes requires further validation through well-designed prospective studies with standardized methodologies.

### Recommendation

The relationship between thyroid hormones and the coagulation system is still underrecognized in clinical practice. Although changes in fibrinogen and D-dimer levels have been reported in patients with thyroid dysfunction, their exact clinical importance is not yet fully understood. These findings highlight the need for greater awareness that thyroid disorders can affect the balance between coagulation and fibrinolysis. Further well-designed prospective studies and systematic reviews are needed to better understand these relationships and determine the clinical value of these biomarkers in predicting hemostatic complications.

### Limitations

This review has some limitations. The included studies varied in their design, study populations, definitions of thyroid dysfunction, and assessment of bleeding and thrombotic outcomes, which may affect direct comparison of findings. Differences in laboratory methods and measurement techniques for fibrinogen and D-dimer may also have contributed to variation between studies. In addition, differences in disease severity, treatment status, and duration of thyroid dysfunction may influence hemostatic changes. Finally, because this review was based on published studies, publication bias cannot be excluded, as studies with negative or non-significant findings may be less likely to be reported.

## Data Availability

No datasets were generated or analysed during the current study.

## Abbreviations

D-dimer: D-dimer
DVT: Deep Vein Thrombosis
FVIII: Factor VIII
IL: Interleukin
LT4: Levothyroxine
PAI-1: Plasminogen Activator Inhibitor
PE: Pulmonary Embolism
SCH: Subclinical Hypothyroidism
TSH: Thyroid Stimulating Hormone
TSH: Thyroid Stimulating Hormone
VTE: Vein Thrombosis
VWF: Von Willebrand factor
